# Nitric oxide reversibly binds the reduced [2Fe-2S] cluster in mitochondrial outer membrane protein mitoNEET and inhibits its electron transfer activity

**DOI:** 10.3389/fmolb.2022.995421

**Published:** 2022-09-07

**Authors:** Chelsey R. Fontenot, Zishuo Cheng, Huangen Ding

**Affiliations:** Department of Biological Sciences, Louisiana State University, Baton Rouge, LA, United States

**Keywords:** nitric oxide, iron-sulfur, electron transfer (ET), oxidation reduction, mitochondia

## Abstract

MitoNEET is a mitochondrial outer membrane protein that regulates energy metabolism, iron homeostasis, and production of reactive oxygen species in cells. Aberrant expression of mitoNEET in tissues has been linked to type II diabetes, neurodegenerative diseases, and several types of cancer. Structurally, the N-terminal domain of mitoNEET has a single transmembrane alpha helix that anchors the protein to mitochondrial outer membrane. The C-terminal cytosolic domain of mitoNEET hosts a redox active [2Fe-2S] cluster via an unusual ligand arrangement of three cysteine and one histidine residues. Here we report that the reduced [2Fe-2S] cluster in the C-terminal cytosolic domain of mitoNEET (mitoNEET_45-108_) is able to bind nitric oxide (NO) without disruption of the cluster. Importantly, binding of NO at the reduced [2Fe-2S] cluster effectively inhibits the redox transition of the cluster in mitoNEET_45-108_. While the NO-bound [2Fe-2S] cluster in mitoNEET_45-108_ is stable, light excitation releases NO from the NO-bound [2Fe-2S] cluster and restores the redox transition activity of the cluster in mitoNEET_45-108_. The results suggest that NO may regulate the electron transfer activity of mitoNEET in mitochondrial outer membrane via reversible binding to its reduced [2Fe-2S] cluster.

## 1 Introduction

Nitric oxide (NO) is a signaling molecule in cardiovascular and neuronal systems (Denninger and Marletta, 1999; Hanafy et al., 2001) and an effective weapon to kill pathogenic bacteria and tumor cells (Bogdan, 2001; Weiss and Schaible, 2015). Because of its chemical nature, NO has a high reactivity with ferrous iron in hemes and iron-sulfur clusters in proteins (Brown, 2007). The interactions between NO and hemes in proteins have been extensively investigated (Denninger and Marletta, 1999; Hanafy et al., 2001; Montfort et al., 2017). However, much less has been known on the interactions between NO and iron-sulfur clusters in proteins. When *Escherichia coli* cells are exposed to NO at micromolar concentrations under anaerobic conditions, a large number of iron-sulfur proteins in the cells are modified forming the protein-bound dinitrosyl iron complex (Ren et al., 2008). *In vitro* studies have also demonstrated that iron-sulfur clusters in proteins can be modified by NO forming dinitrosyl iron complex (Kennedy et al., 1997; Ding and Demple, 2000; Landry et al., 2011), thiolate-bridged diiron tetranitrosyl complex (Tinberg et al., 2010), or octa-nitrosyl cluster (Crack et al., 2011), depending on specific iron-sulfur proteins, NO concentrations, and other experimental conditions.

MitoNEET (also known as CISD1, C10orf70, MDS029, MGC14684, and ZCD1) is the first iron-sulfur protein found in mitochondrial outer membrane (Colca et al., 2004). The N-terminal domain of mitoNEET has a single transmembrane alpha-helix that anchors the protein to mitochondrial outer membrane (Colca et al., 2004). The C-terminal cytosolic domain of mitoNEET binds a redox active [2Fe-2S] cluster via an unusual ligand arrangement of three cysteine and one histidine residues (Hou et al., 2007; Lin et al., 2007; Paddock et al., 2007; Conlan et al., 2009a). Increasing evidence suggests that mitoNEET is the key regulator of energy metabolism, production of free radicals, and iron homeostasis in cells (Tamir et al., 2015; Mittler et al., 2019). Aberrant expression of mitoNEET in tissues has been associated with type II diabetes (Geldenhuys et al., 2021), neurodegenerative diseases (Geldenhuys et al., 2017), and several types of cancer (Sohn et al., 2013; Bai et al., 2015). In addition to mitoNEET, there are two mitoNEET-related proteins in mitochondria: Miner1 (also known as CISD2, ERIS, WFS2, ZCD2, and NAF-1) (Conlan et al., 2009b) and Miner2 (also known as CISD3 and MiNT) (Lipper et al., 2018). Like mitoNEET, both Miner1 and Miner2 bind [2Fe-2S] clusters via three cysteine and one histidine residues. Miner 1 is a homolog of mitoNEET that localizes in both mitochondrial outer membrane and endoplasmic reticulum membrane (Sohn et al., 2013), while Miner2 has very little sequence similarity with mitoNEET and is a soluble mitochondrial matrix protein (Cheng et al., 2017; Lipper et al., 2018). The redox midpoint potential of the [2Fe-2S] cluster in mitoNEET is about −30 mV at pH 7.4 (Bak et al., 2009). Because the intracellular redox potential in mammalian cells is around −360 mV (Hanson et al., 2004), the [2Fe-2S] cluster in mitoNEET is most likely in a reduced state in cells under normal physiological conditions (Landry and Ding, 2014).

**FIGURE 1:**
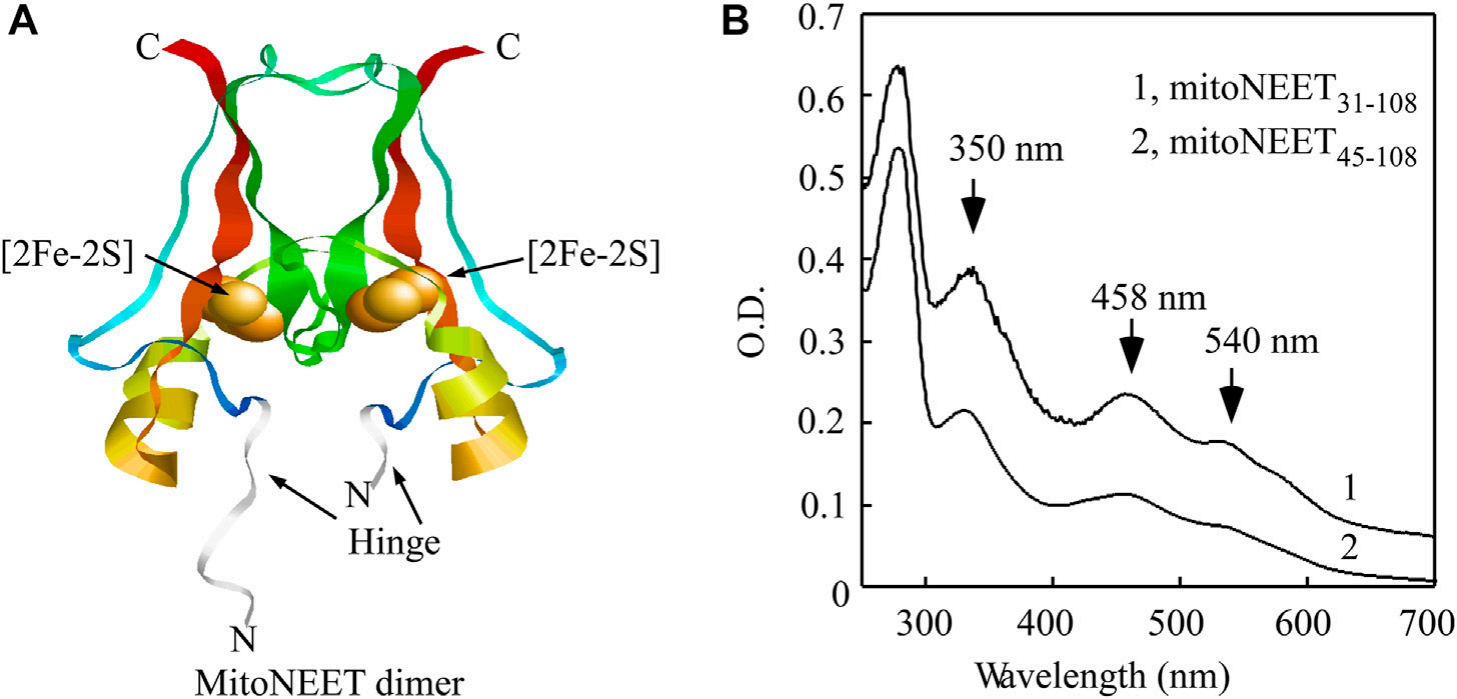
Binding of the [2Fe-2S] cluster in the C-terminal domain of mitoNEET (mitoNEET_45-108_). **(A)**, crystal structure model of the soluble mitoNEET (mitoNEET_31-108_) (PDB code: 2QD0 (Lin et al., 2007),). The flexible hinge region in mitoNEET_31-108_ is shown in grey. The figure was generated using RASMOL (Sayle and Milner-White, 1995). **(B)**, UV-Vis absorption spectra of soluble mitoNEET (mitoNEET_31-108_) and the C-terminal domain of mitoNEET (mitoNEET_45-108_) purified from *E. coli* cells. Purified protein was in buffer containing NaCl (500 mM) and Tris (20 mM, pH 8.0).

Since NO has a regulatory role for mitochondrial functions (Brown, 2007), we have postulated that NO may modulate the specific activities of mitoNEET and its related proteins, Miner1 and Miner2, in mitochondria by directly interacting with the [2Fe-2S] clusters in these proteins. Previous studies have shown that unlike other iron-sulfur proteins (Kennedy et al., 1997; Ding and Demple, 2000; Landry et al., 2011), the reduced [2Fe-2S] clusters in Miner2 can reversibly bind NO without disruption of the clusters (Cheng et al., 2017; Wang et al., 2019). Interestingly, the reduced [2Fe-2S] cluster in the soluble mitoNEET (mitoNEET_31-108_) fails to bind NO (Cheng et al., 2017). The crystallographic studies indicated that in the soluble mitoNEET_31-108_, the hinge region (residues 31–44) that links the C-terminal cytosolic domain and the N-terminal transmembrane domain is highly flexible (Figure 1A) (Conlan et al., 2009a) and potentially blocks NO access to the reduced [2Fe-2S] cluster in mitoNEET_31-108_ without the hinge region 31-44. Here we find that the reduced [2Fe-2S] cluster in the C-terminal domain of mitoNEET (mitoNEET_45-108_) has the same NO binding activity as the reduced [2Fe-2S] clusters in Miner2. Importantly, binding of NO at the reduced [2Fe-2S] cluster effectively inhibits the redox transition activity of the cluster in mitoNEET_45-108_. While the NO-bound [2Fe-2S] cluster in mitoNEET_45-108_ is stable, light excitation releases NO from the NO-bound [2Fe-2S] cluster and restores the redox transition activity of the cluster in mitoNEET_45-108_. The results suggest that NO may regulate the electron transfer activity of mitoNEET in mitochondrial outer membrane via reversible binding to the reduced [2Fe-2S] cluster in the protein.

## 2 Materials and methods

### 2.1 Purification of the soluble mitoNEET (mitoNEET_31-108_) and the C-terminal domain of mitoNEET (mitoNEET_45-108_)

The genes encoding the soluble mitoNEET (mitoNEET_31-108_) (residues 31–108) and the C-terminal domain of mitoNEET (mitoNEET_45-108_) (residues 45–108) were synthesized (GenScript co.) and cloned into pET28b^+^ for protein expression. The sequence of mitoNEET_31-108_ is as following: MERFYVKDHRNKAMINLHIQKDNPKIVHAFDMEDLGDK AVYCRCWRSKKFPFCDGAHTKHNEETGDNVGPLIIKKKET KLAAALEHHHHHH. The C-terminal domain of mitoNEET (mitoNEET_45-108_) does not contain the hinge region (underlined residues 31–44). Both mitoNEET_31-108_ and mitoNEET_45-108_ were produced in *E. coli* BL21 cells and purified as described previously (Li et al., 2018). The purity of purified proteins was greater than 90% as judged by electrophoresis analysis on a 15% polyacrylamide gel containing SDS followed by staining with Coomassie Blue. The protein concentration of purified mitoNEET_31-108_ and mitoNEET_45-108_ was calculated from the amplitude of the absorption peak at 280 nm using the extinction coefficients of 8.61 mM^−1^cm^−1^ and 7.33 mM^−1^cm^−1^, respectively. The UV-visible absorption spectra were recorded in a Beckman DU640 UV-visible spectrometer equipped with a temperature controller.

### 2.2 NO binding and release from the reduced [2Fe-2S] cluster in mitoNEET_45-108_

For the *in vitro* NO binding, purified mitoNEET_31-108_ or mitoNEET_45-108_ in buffer containing NaCl (500 mM) and Tris (20 mM, pH 8.0) was degassed with pure argon (Air co) for 15 min, followed by reduction of the [2Fe-2S] cluster in the protein with freshly prepared dithiothreitol (4 mM) under anaerobic conditions (Landry and Ding, 2014). The NO-saturated solution was prepared as following: nitric oxide (NO) gas (Praxair Co.) was first passed through a soda-lima column to remove NO_2_ and higher nitrogen oxides before being used to bubble pre-degassed double-distilled water in a sealed 50-ml flask for 5 min. The NO concentration in the prepared NO solution was measured using a nitric oxide electrode (World Precision Instrument. co) (Duan et al., 2009). Aliquot of the prepared NO solution was added to the pre-degassed protein samples in a sealed vial using a gas-tight Hamilton syringe, followed by the UV-Vis absorption measurements. For the NO treatment of *E. coli* cells, the exponentially growing *E. coli* cells expressing mitoNEET_45-108_ were concentrated to OD at 600 nm of 20.0, purged with pure argon gas (Air co.) in a sealed flask for 15 min, and treated with diethylamine NONOate (Cayman Chemical co) (75 μM) for 30 min at room temperature under anaerobic conditions (Cheng et al., 2017). After the NO treatment, *E. coli* cells were passed through French Press once, and mitoNEET_45-108_ was purified from the cell extracts as described previously (Li et al., 2018).

For the NO release from the NO-bound [2Fe-2S] cluster in mitoNEET_45-108_, the protein was subjected to light excitation (a broad-spectrum light at 400 K lux) for 5 min under aerobic conditions using a Cole-Parmer 41720 series Illuminator equipped with an infrared filter that reduces the transmittance of heat to the sample. The intensity of the light exposure was measured using a Digital Lux Meter (LX1330B, Dr. Meter).

### 2.3 Electron paramagnetic resonance (EPR) measurements

The X-band EPR spectra were recorded using a Bruker model ESR-300 spectrometer equipped with an Oxford Instruments 910 continuous flow cryostat. Routine EPR conditions: microwave frequency, 9.47 GHz; microwave power, 10.0 mW; modulation frequency, 100 kHz; modulation amplitude, 1.2 mT; temperature, 20 K; receiver gain, 2 × 10^5^.

### 2.4 Redox transition measurements of the [2Fe-2S] cluster in mitoNEET_45-108_

Redox transition of the [2Fe-2S] cluster in mitoNEET_45-108_ was monitored as described previously (Landry et al., 2017). Briefly, mitoNEET_45-108_ (containing 10 μM [2Fe-2S] cluster) was pre-incubated with FMN (0.2 μM) and flavin reductase (0.2 μM) in buffer containing NaCl (500 mM) and Tris (20 mM, pH 8.0) under aerobic condition. The reaction was initiated by adding NADH (50 μM) and monitored by continuously taking UV-Vis absorption spectra of the solution. The amplitude of the absorption peak at 458 nm was used to monitor the redox state of the [2Fe-2S] cluster in mitoNEET_45-108_.

### 2.5 Chemicals

NADH, isopropyl-β-D-1-thiogalactopyranoside, and kanamycin were purchased from Research Product International co. Diethylamine NONOate was purchased from Cayman Chemical. The concentration of diethylamine NONOate was measured in NaOH (10 mM) solution at 250 nm using an extinction coefficient of 6.5 mM^−1^cm^−1^. Half-life time of diethylamine NONOate at pH 7.4 solution is about 16 min. Each diethylamine NONOate molecule releases about 1.5 equivalents of NO (Cayman Chemical co). FMN and other chemicals were purchased from Sigma co. The extinction coefficients of 6.2 mM^−1^cm^−1^ at 340 nm and 12.5 mM^−1^cm^−1^ at 445 nm were used for determining the concentrations of NADH and FMN, respectively (Heikal, 2010). *E. coli* flavin reductase (encoded by gene *fre*) was prepared as described previously (Landry et al., 2017).

## 3 Results

### 3.1 The reduced [2Fe-2S] cluster in the C-terminal domain of mitoNEET (mitoNEET_45-108_) binds nitric oxide (NO) without disruption of the cluster

Previously, we reported that the reduced [2Fe-2S] cluster in the mitoNEET-related protein 2 (Miner2) (Lipper et al., 2018) can reversibly bind nitric oxide (NO) without disruption of the cluster (Cheng et al., 2017; Wang et al., 2019). On the other hand, the reduced [2Fe-2S] cluster in the soluble mitoNEET (mitoNEET_31-108_) fails to bind NO. However, when mitoNEET_31-108_ is denatured using 6 M urea, the reduced [2Fe-2S] cluster in the soluble mitoNEET (mitoNEET_31-108_) can bind NO (Cheng et al., 2017). In the crystal structure of the soluble mitoNEET (mitoNEET_31-108_) (Hou et al., 2007; Lin et al., 2007; Paddock et al., 2007; Conlan et al., 2009a), the hinge region (residues 31–44) that links the C-terminal cytosolic domain and the N-terminal transmembrane *α*-helix domain is highly flexible (Figure 1A). It is likely that the hinge region in native mitoNEET may be not so flexible due to the N-terminal transmembrane alpha-helix domain. To test the idea that the flexible hinge region may block NO access to the reduced [2Fe-2S] cluster in the soluble mitoNEET (mitoNEET_31-108_), we deleted the hinge region (residues 31–44) from the soluble mitoNEET and created the C-terminal domain of mitoNEET (mitoNEET_45-108_). Both mitoNEET_31-108_ and mitoNEET_45-108_ were expressed in *E. coli* cells and purified. The UV-Vis absorption measurements showed that mitoNEET_31-108_ and mitoNEET_45-108_ had the same absorption peaks at 350 nm, 458 nm, and 540 nm, representing the oxidized [2Fe-2S] cluster in the protein (Figure 1B), indicating that deletion of the hinge region does not affect the [2Fe-2S] cluster binding in mitoNEET_45-108_.

**FIGURE 2:**
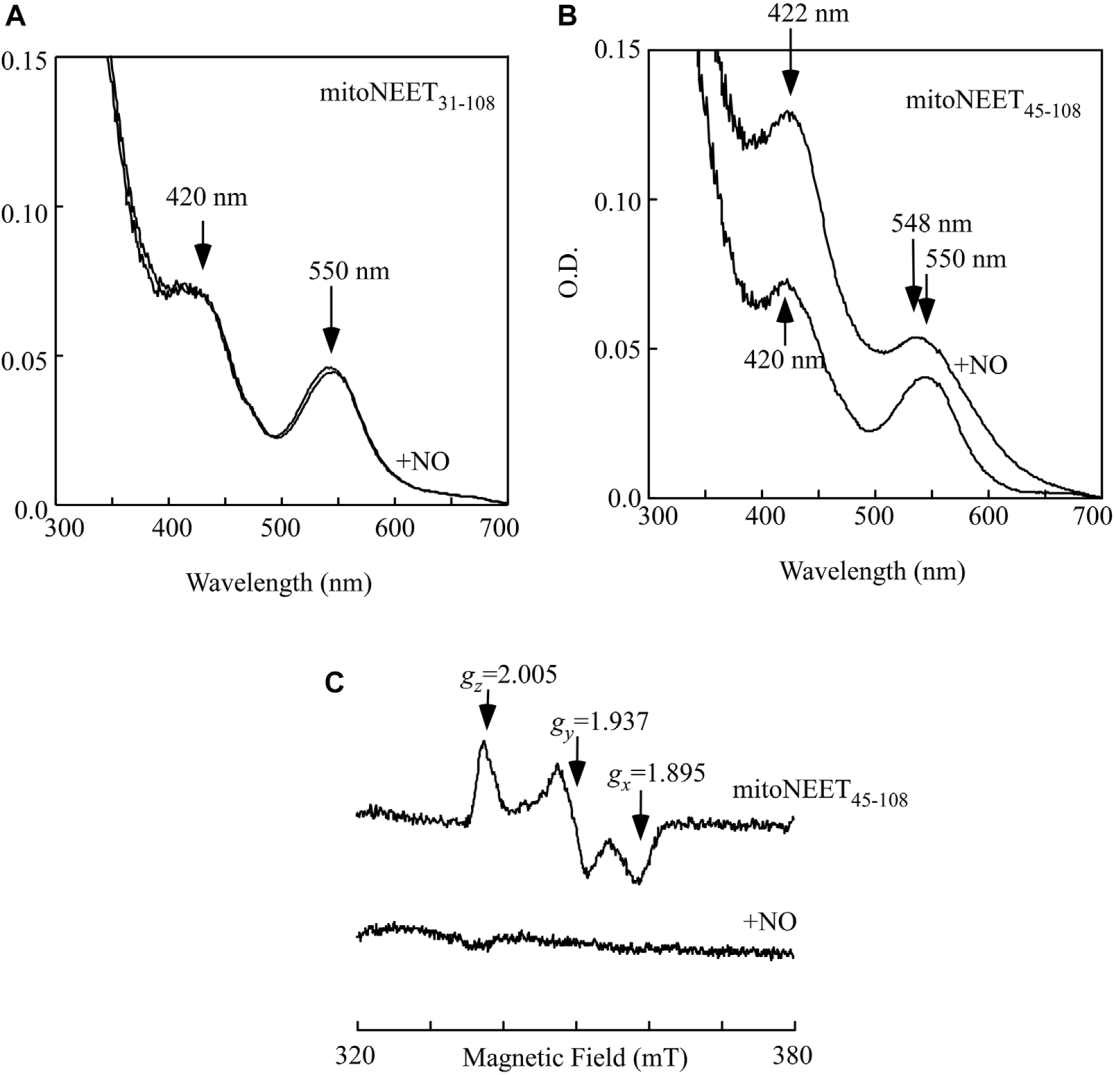
NO binding at the reduced [2Fe-2S] cluster in mitoNEET_45-108_ *in vitro*. **(A)**, purified mitoNEET_31-108_ (30 μM) was reduced with dithiothreitol (4 mM) under anaerobic conditions. An aliquot of NO solution was added into the reaction solution anaerobically. The final concentration of NO added into the reaction solution was about 60 μM. Spectrum 1, the reduced [2Fe-2S] cluster in mitoNEET_31-108_. Spectrum 2, the reduced [2Fe-2S] cluster in mitoNEET_31-108_ after addition of NO. **(B)**, purified mitoNEET_45-108_ (30 μM) was reduced with dithiothreitol (4 mM) under anaerobic conditions. An aliquot of NO solution was added into the reaction solution anaerobically. The final concentration of NO added into the reaction solution was about 60 μM. Spectrum 1, the reduced [2Fe-2S] cluster in mitoNEET_45-108_. Spectrum 2, the reduced [2Fe-2S] cluster in mitoNEET_45-108_ after addition of NO. **(C)**, EPR spectra of the reduced [2Fe-2S] cluster in mitoNEET_45-108_ before and after addition of NO. Purified mitoNEET_45-108_ (30 μM) was reduced with dithiothreitol (4 mM) (spectrum 1), followed by addition of NO (60 μM) (spectrum 2). The data are representatives of three independent experiments.

The soluble mitoNEET (mitoNEET_31-108_) and the C-terminal domain of mitoNEET (mitoNEET_45-108_) were then reduced with dithiothreitol (4 mM) under anaerobic conditions as described previously (Landry and Ding, 2014). Figure 2A shows that reduction of the [2Fe-2S] cluster in the soluble mitoNEET (mitoNEET_31-108_) resulted in a new absorption spectrum with two dominant absorption peaks at 420 and 550 nm (spectrum 1). Addition of two-fold excess of NO under anaerobic conditions had very little or no effect on the absorption peaks at 420 and 550 nm (spectrum 2), indicating that the reduced [2Fe-2S] cluster in mitoNEET_31-108_ fails to bind NO as reported previously (Cheng et al., 2017). In contrast, when the reduced [2Fe-2S] cluster in the C-terminal domain of mitoNEET (mitoNEET_45-108_) was treated with two-fold excess of NO under anaerobic conditions, a new absorption spectrum appeared with shift of the absorption peaks at 420 nm to 422 nm and 550 nm to 548 nm (Figure 2B), indicating the NO binding at the reduced [2Fe-2S] cluster in mitoNEET_45-108_. The NO binding at the reduced [2Fe-2S] cluster in mitoNEET_45-108_ was further confirmed by using the electrospray ionization mass spectrometry (see Supplementary Figure S1), as described previously for Miner2 (Cheng et al., 2017).

While the oxidized [2Fe-2S] cluster in mitoNEET is diamagnetic and has no electron paramagnetic resonance (EPR) signal, the reduced [2Fe-2S] cluster in mitoNEET has the distinct EPR signals at *g*_*x*_ = 1.859, *g*_*y*_ = 1.937, and *g*_*z*_ = 2.005 (Iwasaki et al., 2009; Landry et al., 2017) (Figure 2C, spectrum 1). When the reduced [2Fe-2S] cluster in mitoNEET_45-108_ was treated with two-fold excess of NO under anaerobic conditions, the EPR signals at *g*_*x*_ = 1.859, *g*_*y*_ = 1.937, and *g*_*z*_ = 2.005 largely disappeared (Figure 2C, spectrum 2), as reported previously for the reduced [2Fe-2S] cluster in Miner2 (Cheng et al., 2017). The results suggest that the reduced [2Fe-2S] cluster in mitoNEET_45-108_, like that in Miner2 (Cheng et al., 2017), binds NO and becomes the EPR silent.

### 3.2 The reduced [2Fe-2S] cluster in mitoNEET_45-108_ expressed in *E. coli* cells can also bind NO

**FIGURE 3:**
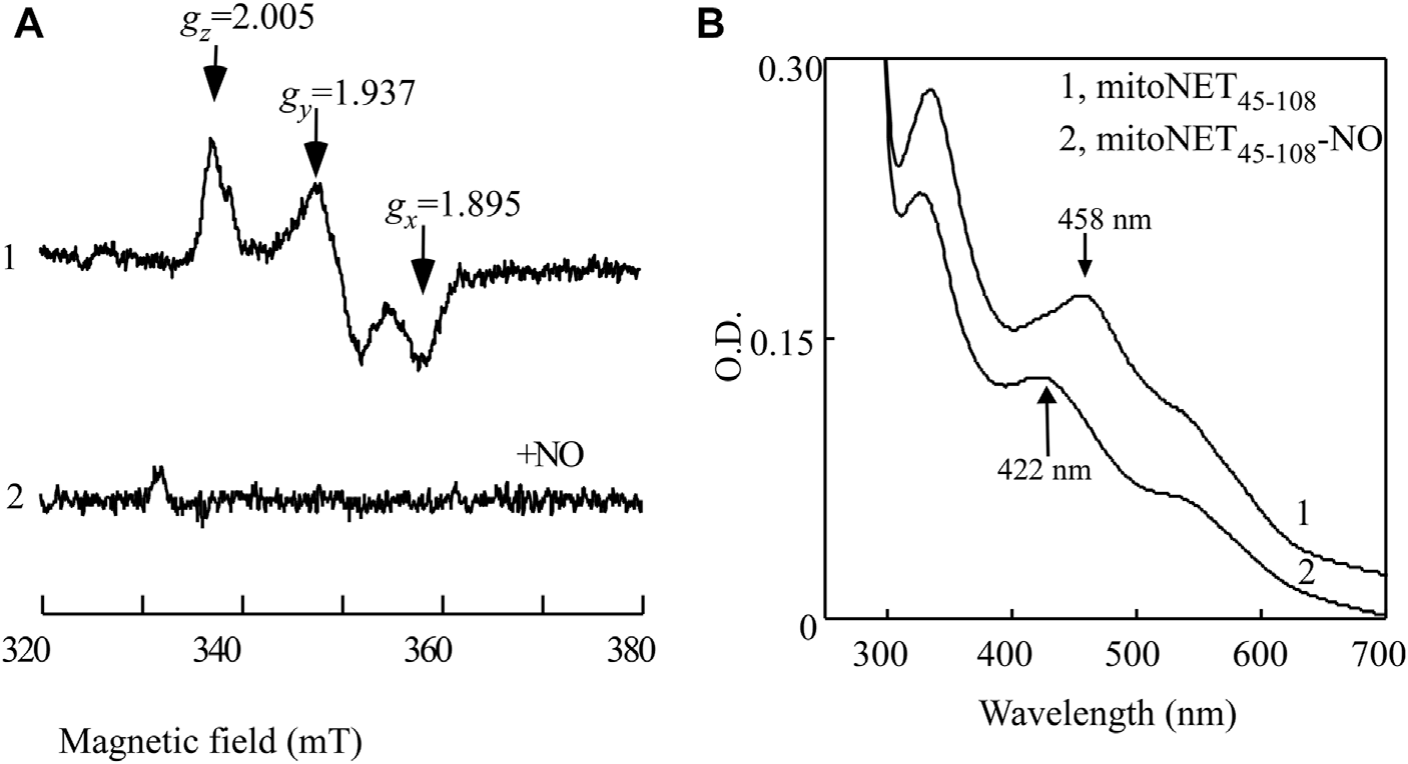
NO binding at the reduced [2Fe-2S] cluster in mitoNEET_45-108_ expressed in *E. coli* cells. **(A)**, EPR spectra of the *E. coli* cells expressing mitoNEET_45-108_ treated with or without NO. The intact *E. coli* cells expressing mitoNEET_45-108_ were concentrated to O.D. at 600 nm of 20. The cells were treated with or without NO donor (75 μM NONOate) at room temperature for 30 min under anaerobic conditions. Aliquots (300 μL) of the cells were transferred to EPR tubes and immediately frozen in liquid nitrogen until the EPR measurements. The EPR spectra of the *E. coli* cells without recombinant mitoNEET_45-108_ were used as the baseline. Spectrum 1, EPR spectrum of the mitoNEET_45-108_ in *E. coli* cells. Spectrum 2, EPR spectrum of the mitoNEET_45-108_ in *E. coli* cells after the NO exposure. **(B)**, UV-Vis absorption spectra of mitoNEET_45-108_ purified from the *E. coli* cells treated with or without the NO donor. Spectrum 1, mitoNEET_45-108_ purified from the *E. coli* cells without the NO treatment. Spectrum 2, mitoNEET_45-108_ purified from the *E. coli* cells treated with NO donor. The data are the representatives from three independent experiments.

When mitoNEET_45-108_ was expressed in *E. coli* cells, the whole-cell EPR measurements showed that the [2Fe-2S] cluster of mitoNEET_45-108_ was in a reduced state with the EPR signals at *g*_*x*_ = 1.859, *g*_*y*_ = 1.937, and *g*_*z*_ = 2.005 (Figure 3A, spectrum 1). When the *E. coli* cells expressing mitoNEET_45-108_ were treated with the NO donor (NONOate) under anaerobic conditions, the EPR signals of the reduced [2Fe-2S] cluster of mitoNEET_45-108_ largely disappeared (Figure 3A, spectrum 2), indicating binding of NO to the reduced [2Fe-2S] cluster in mitoNEET_45-108_ in the cells. MitoNEET_45-108_ was then purified from the *E. coli* cells treated with or without NO. Figure 3B shows that without the NO treatment, purified mitoNEET_45-108_ had the oxidized [2Fe-2S] cluster with a major absorption peak at 458 nm. The NO treatment of the *E. coli* cells resulted in the mitoNEET_45-108_ with an absorption peak at 422 nm, indicative of the NO-bound [2Fe-2S] cluster in the protein. Thus, the reduced [2Fe-2S] cluster in mitoNEET_45-108_ can bind NO not only *in vitro* (Figure 2) but also in *E. coli* cells (Figure 3).

### 3.3 Binding of NO at the reduced [2Fe-2S] cluster in mitoNEET_45-108_ inhibits the redox transition of the cluster

In previous studies, we reported that the soluble mitoNEET (mitoNEET_31-108_) acts as a novel redox enzyme that can transfer electrons from the reduced flavin mononucleotide (FMNH_2_) to oxygen or ubiquinone via redox transition of the [2Fe-2S] cluster (Landry et al., 2017; Wang et al., 2017; Li et al., 2018; Tasnim et al., 2020; Tasnim and Ding, 2022). Together with flavin reductase that reduces FMN to FMNH_2_ using NADH as electron donors, mitoNEET promotes oxidation of NADH and reduction of oxygen or ubiquinone (Wang et al., 2017; Tasnim et al., 2020; Tasnim and Ding, 2022). Here, we asked whether binding of NO at the reduced [2Fe-2S] cluster will affect the redox transition of the cluster in mitoNEET_45-108_.

**FIGURE 4:**
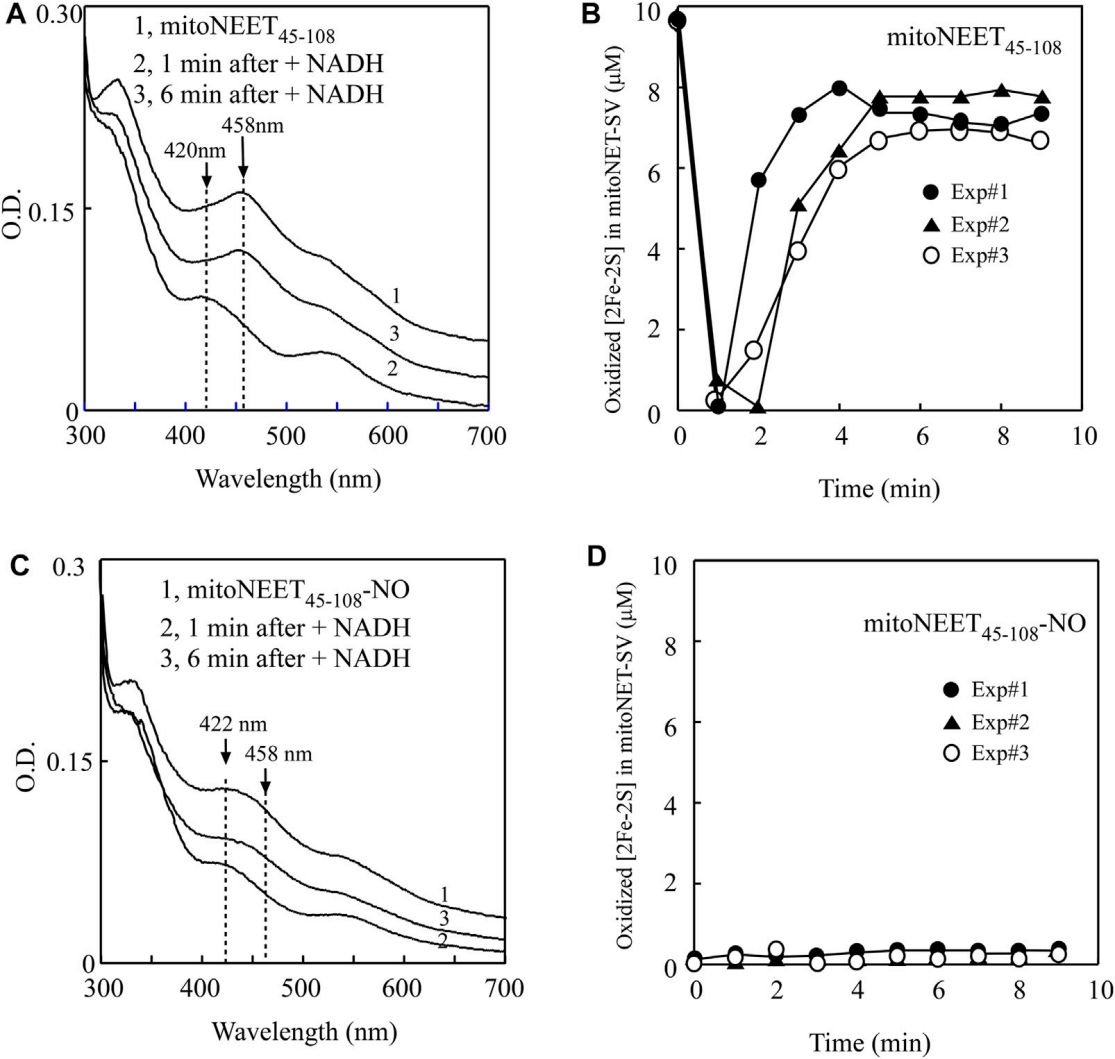
NO binding at the reduced [2Fe-2S] cluster in mitoNEET_45-108_ inhibits the redox transition of the cluster. **(A)**, the redox transition of the [2Fe-2S] cluster in mitoNEET_45-108_. Spectrum 1, mitoNEET_45-108_ (10 μM) was pre-incubated with FMN (0.2 μM) and flavin reductase (0.2 μM) under aerobic conditions. The absorption peaks at 420 and 458 nm indicate the reduced and oxidized [2Fe-2S] cluster in mitoNEET_45-108_, respectively. Spectra 2 and 3 were taken at 1 and 6 min after addition of NADH (50 μM) to the reaction solution, respectively. **(B)**, time course of redox transition of the [2Fe-2S] cluster in mitoNEET_45-108_. The amplitude of the absorption peak at 458 nm was used to quantify the oxidized [2Fe-2S] cluster in mitoNEET_45-108_. The amounts of the oxidized [2Fe-2S] cluster in mitoNEET_45-108_ were plotted as a function of the time after addition of NADH. Closed circles, closed triangles, and open circles represent three independent experiment results. **(C)**, the redox transition of the NO-bound [2Fe-2S] cluster in mitoNEET_45-108_. Spectrum 1, the NO-bound mitoNEET_45-108_ (10 μM) was pre-incubated with FMN (0.2 μM) and flavin reductase (0.2 μM) under aerobic conditions. The absorption peak at 422 nm indicates the NO-bound [2Fe-2S] cluster in mitoNEET_45-108_. Spectra 2 and 3 were taken at 1 and 6 min after addition of NADH (50 μM), respectively. **(D)**, time course of the redox transition of the NO-bound [2Fe-2S] cluster in mitoNEET_45-108_. The amounts of the oxidized [2Fe-2S] cluster in mitoNEET_45-108_ were plotted as a function of the time after addition of NADH. Closed circles, closed triangles, and open circles represent three independent experiment results.

For the experiments, purified mitoNEET_45-108_ was pre-incubated with catalytic amounts of FMN and flavin reductase under aerobic conditions. The reaction was initiated by adding NADH to the solution. The redox transition of the [2Fe-2S] cluster in mitoNEET_45-108_ was monitored by continuously taking the absorption spectra. Figure 4A shows that the oxidized [2Fe-2S] cluster (the absorption peak at 458 nm) of mitoNEET_45-108_ (spectrum 1) was quickly reduced (the absorption peak at 420 nm) upon addition of NADH (spectrum 2), followed by re-oxidation (spectrum 3) due to oxygen in the solution. The time course of the redox transition of the [2Fe-2S] cluster in mitoNEET_45-108_ (from three independent experiments) is shown in Figure 4B. The results suggest that the [2Fe-2S] cluster in mitoNEET_45-108_, like that in mitoNEET_31-108_ (Wang et al., 2017), can undergo the redox transition in the presence of FMN, flavin reductase, and NADH under aerobic conditions.

In parallel experiments, the NO-treated mitoNEET_45-108_ was also pre-incubated with catalytic amounts of FMN and flavin reductase under aerobic conditions. Figure 4C shows that the NO-bound [2Fe-2S] cluster in mitoNEET_45-108_ did not have any redox transition after addition of NADH to the reaction solution. The time course of the redox transition of the NO-bound [2Fe-2S] cluster in mitoNEET_45-108_ is shown in Figure 4D. Thus, the NO binding at the reduced [2Fe-2S] cluster effectively inhibits the redox transition of the cluster in mitoNEET_45-108_.

### 3.4 Light excitation releases NO from the NO-bound [2Fe-2S] cluster in mitoNEET_45-108_ and restores the redox transition activity of the cluster

**FIGURE 5:**
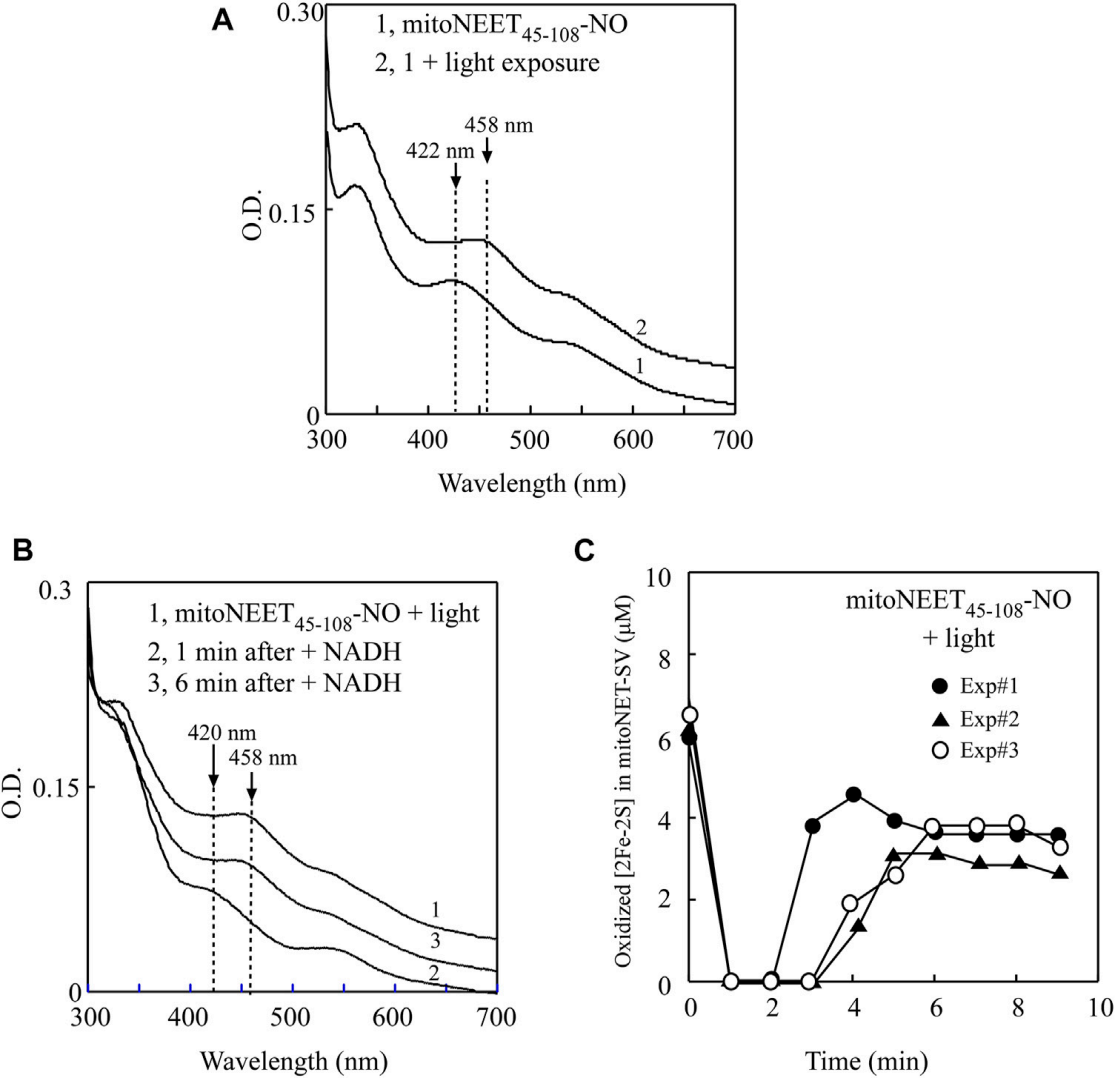
Light exposure releases NO from the NO-bound [2Fe-2S] cluster in mitoNEET_45-108_ and restores the redox transition activity of the cluster. **(A)**, UV-Vis absorption spectra of the NO-bound mitoNEET_45-108_ before and after light exposure. The NO-bound [2Fe-2S] cluster in mitoNEET_45-108_ (∼6 μM) (spectrum 1) was exposed to light (400K lux) for 5 min (Spectrum 2). The absorption peaks at 422 and 458 nm represent the NO-bound [2Fe-2S] cluster and the oxidized [2Fe-2S] cluster in mitoNEET_45-108_, respectively. **(B)**, the redox transition of the [2Fe-2S] cluster in the NO-bound [2Fe-2S] cluster in mitoNEET_45-108_ after the light exposure. Spectrum 1, the NO-bound [2Fe-2S] cluster in mitoNEET_45-108_ after the light exposure was pre-incubated with FMN (0.2 μM) and flavin reductase (0.2 μM) under aerobic conditions. Spectra 2 and 3 were taken at 1 and 6 min after addition of NADH (50 μM) to the reaction solution, respectively. **(C)**, time course of the redox transition of the NO-bound [2Fe-2S] cluster in mitoNEET_45-108_ after the light exposure. The amounts of the oxidized [2Fe-2S] cluster in mitoNEET_45-108_ were plotted as a function of the time after addition of NADH. Closed circles, closed triangles, and open circles represent three independent experiment results.

While the NO-bound [2Fe-2S] cluster in Miner2 is stable, light excitation quickly releases NO from the NO-bound [2Fe-2S] cluster in the protein (Wang et al., 2019). To further explore the physio-chemical property of the NO-bound [2Fe-2S] cluster in proteins, we subjected the NO-bound [2Fe-2S] cluster in mitoNEET_45-108_ to light excitation. The UV-Vis absorption spectra of the NO-bound [2Fe-2S] cluster in mitoNEET_45-108_ were taken before and after light excitation. Figure 5A shows that the absorption peak at 422 nm of the NO-bound [2Fe-2S] cluster in mitoNEET_45-108_ was shifted to 458 nm of the oxidized [2Fe-2S] cluster after light excitation, indicating that light exposure releases NO from the NO-bound [2Fe-2S] cluster in mitoNEET_45-108_ without disruption of the cluster.

The NO-bound mitoNEET_45-108_ after light excitation was further subjected to the redox transition analysis of the [2Fe-2S] cluster in the presence of catalytic amounts of FMN and flavin reductase under aerobic conditions. As shown in Figure 5B, the oxidized [2Fe-2S] cluster (the absorption peak at 458 nm) (spectrum 1) in the light-exposed mitoNEET_45-108_ was quickly reduced (the absorption peak at 420 nm) upon addition of NADH (spectrum 2), followed by re-oxidation (spectrum 3) by oxygen under aerobic conditions. The time course of the redox transition of the [2Fe-2S] cluster in the light-exposed mitoNEET_45-108_ (from three independent experiments) is shown in Figure 5C, indicating that light excitation largely restores the redox transition activity of the NO-bound [2Fe-2S] cluster in mitoNEET_45-108_.

## 4 Discussion

When iron-sulfur proteins are exposed to NO at micromolar concentrations, iron-sulfur clusters in proteins are often modified forming dinitrosyl iron complex (Kennedy et al., 1997; Ding and Demple, 2000; Landry et al., 2011), thiolate-bridged diiron tetranitrosyl complex (Tinberg et al., 2010), or octa-nitrosyl cluster (Crack et al., 2011). Here we find that the reduced [2Fe-2S] cluster in the C-terminal domain of mitoNEET (mitoNEET_45-108_) can bind NO without disruption of the cluster, and that binding of NO at the reduced [2Fe-2S] cluster effectively inhibits the redox transition of the cluster in mitoNEET_45-108_. Furthermore, light excitation releases NO from the NO-bound [2Fe-2S] cluster in mitoNEET_45-108_ and restores the redox transition activity of the cluster. The results led us to propose that NO may regulate the function of mitoNEET by reversibly binding to its reduced [2Fe-2S] cluster.

**FIGURE 6:**
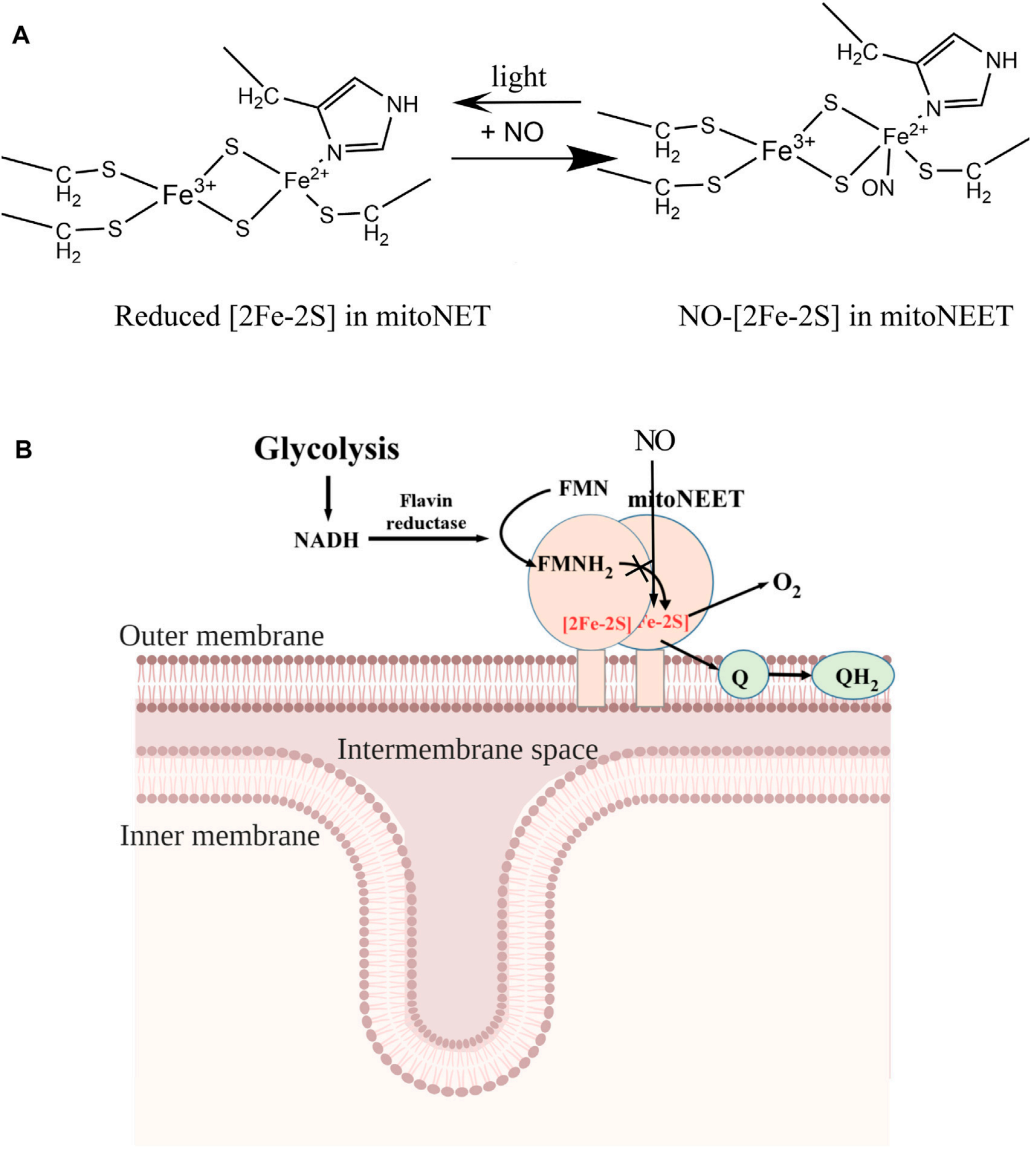
A proposed model for the NO-mediated regulation of the electron transfer activity of mitoNEET. **(A)**, NO binding at the reduced [2Fe-2S] cluster in mitoNEET. MitoNEET binds the [2Fe-2S] cluster via three cysteine and one histidine residues. The ferrous iron (Fe^2+^) of the reduced [2Fe-2S] cluster in mitoNEET is ligated via Cys-83 and His-87, and binds NO. Light excitation releases NO from the NO-bound [2Fe-2S] cluster in mitoNEET. **(B)**, together with flavin reductase and FMN, mitoNEET promotes oxidation of NADH and reduction of oxygen or ubiquinone. Because oxidation of NADH is required for the glycolytic activity, mitoNEET may enhance glycolysis via oxidizing NADH. NO inhibits the redox transition of the [2Fe-2S] cluster in mitoNEET via reversibly binding to the reduced [2Fe-2S] cluster and decreases glycolytic activity in cells.

In previous studies, iron-sulfur clusters in proteins are often found to be destroyed by NO (Landry et al., 2011). For example, the iron-sulfur clusters in the bovine heart aconitase (Kennedy et al., 1997), the *E. coli* redox transcription factor SoxR (Ding and Demple, 2000), and the WhiB-like proteins from *Streptomyces coelicolor* and *Mycobacterium tuberculosis* (Crack et al., 2011) are all readily disrupted by NO. In other cases such as xanthine oxidoreductase (Harrison, 2002), iron-sulfur clusters are resistant to NO. Reversible binding of NO at the reduced [2Fe-2S] cluster in the C-terminal domain of mitoNEET (mitoNEET_45-108_) (Figures 4, 5) and in the mitoNEET-related protein Miner2 (Cheng et al., 2017; Wang et al., 2019) is an unprecedented example. This is likely because mitoNEET_45-108_ and Miner2 host the [2Fe-2S] cluster via the unique ligand arrangement of three cysteine and one histidine residues. In mitoNEET, the [2Fe-2S] cluster is ligated by Cys-72, Cys-74, Cys-83, and His-87 (Hou et al., 2007; Lin et al., 2007; Paddock et al., 2007; Conlan et al., 2009a) (Figure 6A). Spectroscopic and local spin model studies have suggested that the iron atom ligated by Cys-83 and His-87 in mitoNEET is redox active (Iwasaki et al., 2009). This notion has been confirmed by the observation that mutation of His-87 to Cys shifts the redox midpoint potential (E_m7_) of the [2Fe-2S] cluster in mitoNEET_31-108_ from 0 mV to −300 mV (Zuris et al., 2010). Thus, when the [2Fe-2S] cluster in mitoNEET_45-108_ is reduced, the iron atom ligated by Cys-83 and His-87 is in a reduced state for NO binding (Figure 6A). It should be pointed out that the oxidized [2Fe-2S] cluster in mitoNEET_45-108_ or Miner2 does not bind NO, supporting the notion that NO has a strong binding activity for ferrous iron in protein (Brown, 2007). Interestingly, when the NO-bound [2Fe-2S] cluster in mitoNEET_45-108_ is exposed to light excitation, NO is released from the [2Fe-2S] cluster without disruption of the cluster (Figure 5), as reported for the NO-bound [2Fe-2S] cluster in Miner2 (Cheng et al., 2017; Wang et al., 2019). Thus, mitoNEET and Miner2 may represent a new group of iron-sulfur proteins that can reversibly bind NO without disruption of the cluster.

The soluble mitoNEET (mitoNEET_31-108_) has been widely used for biochemical (Zuris et al., 2011; Banci et al., 2015; Lipper et al., 2015; Golinelli-Cohen et al., 2016; Camponeschi et al., 2017; Saralkar et al., 2021; Song et al., 2021; Tasnim and Ding, 2022) and crystallographic studies (Hou et al., 2007; Lin et al., 2007; Paddock et al., 2007; Conlan et al., 2009a). However, native mitoNEET is a membrane-bound protein that contains the N-terminal transmembrane domain (residues 10–31), the hinge region (residues 31–44), and the C-terminal cytosolic domain (residues 45–108). The soluble mitoNEET (mitoNEET_31-108_) contains the C-terminal cytosolic domain and the highly flexible hinge region (residues 31–44) (Conlan et al., 2009a). It is conceivable that the flexible hinge region may block NO access to the reduced [2Fe-2S] cluster in the soluble mitoNEET (mitoNEET_31-108_) (Figure 1A). Indeed, removal of the hinge region facilitates the NO binding at the reduced [2Fe-2S] cluster in the C-terminal domain of mitoNEET (mitoNEET_45-108_). In native mitoNEET, the hinge region may be not as flexible as that in the soluble mitoNEET_31-108_, thus allowing NO binding at the reduced [2Fe-2S] cluster in mitoNEET. It should also be pointed out that there are at least five potential phosphorylation sites in native mitoNEET according to the sequence-based prediction (Blom et al., 1999), and phosphorylation of these sites may also contribute to the conformation change of native mitoNEET in such that the reduced [2Fe-2S] cluster in mitoNEET is accessible to NO.

MitoNEET has been shown to regulate energy metabolism, production of free radicals, and iron homeostasis in cells (Tamir et al., 2015; Mittler et al., 2019). However, the exact function of mitoNEET remains elusive. One hypothesis stated that mitoNEET may act as an iron-sulfur cluster carrier that transports the iron-sulfur clusters assembled in mitochondria to target proteins in cytosol and nucleus (Zuris et al., 2011; Banci et al., 2015; Lipper et al., 2015; Golinelli-Cohen et al., 2016; Camponeschi et al., 2017). However, the cluster transfer between mitoNEET and its partner proteins occurs only when the [2Fe-2S] cluster in mitoNEET is oxidized (Zuris et al., 2011; Banci et al., 2015; Lipper et al., 2015; Golinelli-Cohen et al., 2016; Camponeschi et al., 2017) and the transfer process often takes several hours to complete *in vitro* (Zuris et al., 2011). Since the [2Fe-2S] cluster in mitoNEET is in a reduced state in cells under normal physiological conditions (Bak et al., 2009), specific function of mitoNEET in iron-sulfur cluster biogenesis remains to be further investigated. On the other hand, the [2Fe-2S] cluster in mitoNEET is redox active and can be reduced by FMNH_2_ and oxidized by oxygen or ubiquinone (Landry et al., 2017; Wang et al., 2017; Li et al., 2018; Tasnim et al., 2020; Tasnim and Ding, 2022). These observations led us to propose that mitoNEET may act as a redox enzyme that catalyzes the electron transfer from FMNH_2_ to oxygen or ubiquinone in the membrane via its [2Fe-2S] cluster (Figure 6B). Because FMNH_2_ can be reduced by flavin reductase (Cunningham et al., 2000) using NADH as the electron donors, and NAD^+^ is required for glycolysis in cytosol (Hung and Yellen, 2014), mitoNEET may indirectly oxidize NADH in cytosol and promote glycolysis (Wang et al., 2017; Tasnim and Ding, 2022). Since NO has a crucial regulatory function in energy metabolism in cell (Brown, 1999; Piantadosi and Suliman, 2012), we propose that NO may modulate the glycolytic activity in cells by reversibly binding to the reduced [2Fe-2S] cluster in mitoNEET and inhibiting the electron transfer activity of mitoNEET in mitochondrial outer membrane (Figure 6B).

## Data Availability

The original contributions presented in the study are included in the article/Supplementary Material, further inquiries can be directed to the corresponding author.
