# Supplementary material for: Nitric oxide reversibly binds the reduced [2Fe-2S] cluster in mitochondrial outer membrane protein mitoNEET and inhibits its electron transfer activity
Source: Front Mol Biosci. 2022 Sep 7;9:995421. doi: 10.3389/fmolb.2022.995421 (PMC9490426; doi:10.3389/fmolb.2022.995421)

Supplemental Figure 1

Mass spectra of the mitoNEET$_{45\text{-}108}$ [2Fe-2S] cluster. Purified mitoNEET$_{45\text{-}108}$ was reduced with dithiothreitol and treated with two-fold excess of NO under anaerobic conditions. MitoNEET was then re-purified by passing the sample through a High-Trap Desalting column and subjected to ESI-MS analysis. The experiments were performed with an AmaZon speed ETD Ion Trap mass spectrometer (Bruker Daltonics, Billerica, MA, USA). Sample solution was infused via an Apollo II electrospray ion source using a syringe pump at the flow rate of 5 µL/min. MS detection was performed in a full-scan mode in positive ionization enhanced resolution mode with the scan speed of 8100 m/z/s. The parameter settings for ESI-MS were as follows: capillary voltage, 4500 V; end plate offset, –500 V; nebulizer, 8 psi; dry gas, 4 L/min; dry gas temperature, 180 °C. The software used for data processing was Compass DataAnalysis (Bruker Daltonics). Red spectrum, purified mitoNEET$_{45\text{-}108}$. Blue spectrum, purified mitoNEET$_{45\text{-}108}$ after NO treatment. Peak A at 9475.0 Da represents the mitoNEET$_{45\text{-}108}$ [2Fe-2S] cluster (mitoNEET$_{45\text{-}108}$ (9298) + [2Fe-2S] (177)). Peak B at 9505.4 Da indicates the increase of molecular weight by 30.4 Da, representing NO binding at mitoNEET$_{45\text{-}108}$ [2Fe-2S] cluster.

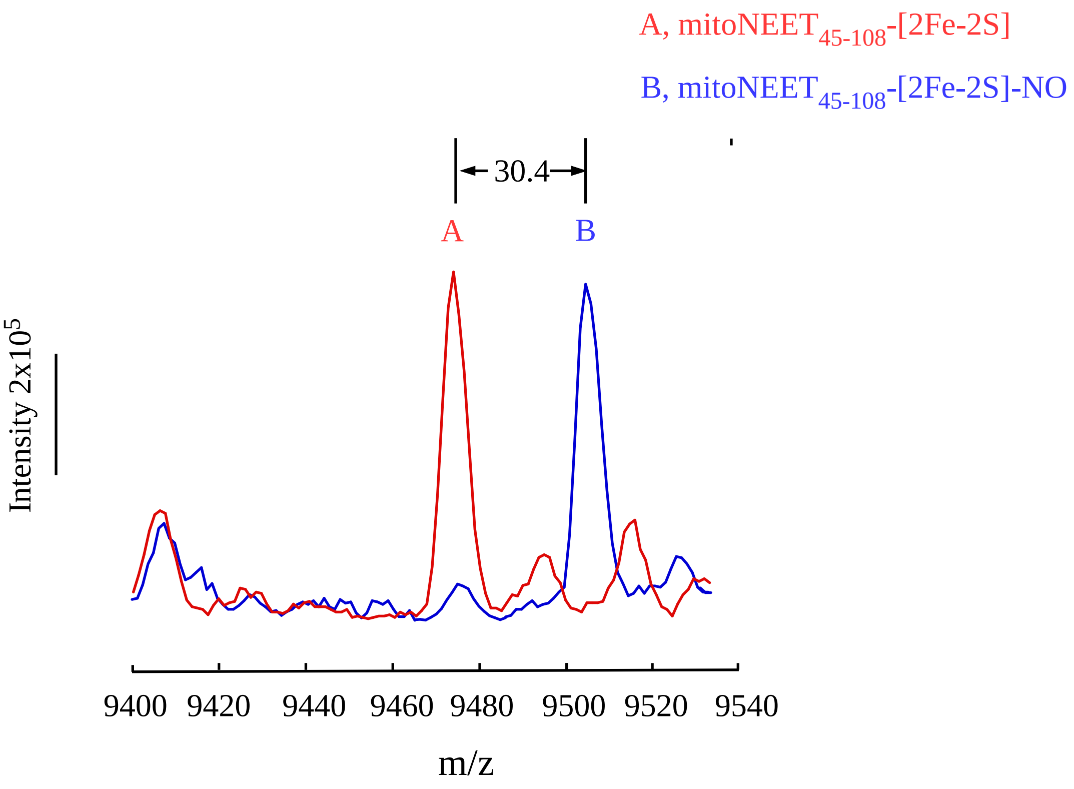

Supplement: Supplementary file 1 [file DataSheet1.PDF]
